# [^68^Ga]Ga-DOTATATE uptake in pheochromocytoma/paraganglioma predicts SSTR2 expression by immunohistochemistry: Strongest uptake in head and neck paraganglioma

**DOI:** 10.1007/s00259-026-07897-0

**Published:** 2026-04-28

**Authors:** Alessa Fischer, Umberto Maccio, Junko Inoue Inukai, Kathrin Zitzmann, Christoph J. Auernhammer, Constanze Hantel, Mercedes Robledo, Friederike Völter, Martin Hüllner, Ashley B. Grossman, Karel Pacak, Felix Beuschlein, Alexander Huber, Stephan Beintner-Skawran, Svenja Nölting

**Affiliations:** 1 Department of Endocrinology, Diabetology and Clinical Nutrition, University Hospital Zurich (USZ) and University of Zurich (UZH), Zurich, 8091 Switzerland; 2 Department of Pathology, University Hospital Zurich (USZ), Zurich, 8091 Switzerland; 3 Department of Nuclear Medicine, University Hospital Zurich (USZ) and University of Zurich (UZH), Zurich, 8091 Switzerland; 4 Department of Radiology, Kobe University Graduate School of Medicine, 7-5-1 Kusunoki-Cho, Chuo-Ku, Kobe, Hyogo 650-0017 Japan; 5 Department of Medicine IV, University Hospital, Ludwig-Maximilians-University Munich, Munich, 80336 Germany; 6 Hereditary Endocrine Cancer Group. Spanish National Cancer Research Center (CNIO), Madrid, Spain; 7 Department of Nuclear Medicine, LMU University Hospital, LMU Munich, Munich, 81377 Germany; 8 Green Templeton College, University of Oxford, Oxford, UK; 9 Center for Adrenal Endocrine Tumors, AKESO, Prague 5, 155 00 Czech Republic; 10 Multidisciplinary Consortium for Adrenal Diseases, Faculty of Medicine and Faculty Hospital, Palacky University, Olomouc, Czech Republic; 11 The LOOP Zurich - Medical Research Center, Zurich, 8044 Switzerland; 12 Department of Otorhinolaryngology, University Hospital Zurich (USZ) and University of Zurich (UZH), Zurich, 8091 Switzerland

**Keywords:** Pheochromocytoma, Head and neck paraganglioma, PPGL, Somatostatin receptor, [^68^Ga]Ga-DOTATATE PET

## Abstract

**Purpose:**

Pheochromocytomas and paragangliomas (PPGLs) are endocrine tumors that may express somatostatin receptors (SSTRs). Importantly, SSTR2 expression is associated with metastatic behavior and succinate dehydrogenase B (*SDHB*) pathogenic variants. Somatostatin receptor analogue (SSA) positron emission tomography-computed tomography (PET/CT) is therefore widely used for diagnosis and localization of PPGLs, particularly in extra-adrenal, metastatic, or *SDHB*-related disease. This study aimed to evaluate whether cellular SSTR expression by immunohistochemistry (IHC) correlates with radionuclide uptake on SSA PET/CT and could serve as a biomarker for detectability on SSA imaging.

**Methods:**

In 35 patients with PPGLs treated at the University Hospital Zurich, IHC for SSTR2 and SSTR5 was performed on tumor tissue samples and evaluated both manually and with digital image analysis (DIA). SSTR2 mRNA expression was determined by qRT-PCR. The association of SSTR expression at the cellular level and standardized uptake value (SUV) on [^68^Ga]Ga-DOTATATE PET/CT was analyzed.

**Results:**

IHC expression was assessed in 38 lesions. Manual and DIA quantification of IHC SSTR2 expression correlated strongly (r_s_ 0.86; *p* < 0.001). Manual SSTR2 expression correlated with both liver-corrected PET maximum SUV (SUV_max_) (r_s_ 0.53, *p* = 0.001) and liver-corrected PET mean SUV (SUV_mean_) (r_s_ 0.48, *p* = 0.002) of the corresponding lesion. SSTR2 expression by IHC was highest in head and neck paragangliomas (HNPGLs), with significantly higher SUV_max_ and SUV_mean_ compared to pheochromocytomas and abdominal paragangliomas. SSTR2 mRNA expression did not correlate with SSTR2 expression by IHC or PET metrics.

**Conclusions:**

SSTR2 expression by IHC correlates with [^68^Ga]Ga-DOTATATE uptake, with highest expression found in HNPGLs.

**Supplementary Information:**

The online version contains supplementary material available at https://doi.org/10.1007/s00259-026-07897-0.

## Introduction

Pheochromocytomas and paragangliomas (together: PPGLs) are relatively rare, endocrine tumors with a high degree of heritability [1]. Pheochromocytomas originate from the adrenal medulla, whereas paragangliomas originate extra-adrenally from paraganglia of the sympathetic or parasympathetic nervous system [2]. PPGLs can produce catecholamines (epinephrine, norepinephrine, or dopamine) in varying proportions, which may lead to typical signs and symptoms of PPGLs [3, 4]. The somatostatin receptors 1–5 (SSTRs 1–5) are G-protein-coupled receptors that bind somatostatin with high affinity [5]. SSTRs are expressed in various (neuro-)endocrine tissues, such as gastro-enteropancreatic neuroendocrine tumors (GEP-NETs) [6, 7]. PPGLs mainly express SSTR2, with higher expression of SSTR2 in metastatic PPGLs and those with succinate dehydrogenase B (*SDHB*) pathogenic variants (PVs) [8–10]. In clinical practice, SSTR2 functional imaging with somatostatin receptor analogue (SSA)-positron emission tomography/computed tomography (PET/CT) is increasingly used for the diagnosis and localization of PPGLs, especially of those with metastatic and *SDHB*-related disease [11]. Gallium-68-labeled [^68^Ga]Ga-DOTATATE PET has proven to be superior to [^18^F]F-DOPA-PET for metastatic paragangliomas and *SDHx*-related PPGLs, while for sporadic metastatic pheochromocytomas and primary non-*SDHx* paragangliomas, both imaging modalities yield similar sensitivity [12, 13]. Binding and uptake of the radionuclide depends on the density of SSTRs on the cell surface and the degree of internalization of the ligand/receptor complex. Hence, the level of SSTR on the cell surface of the PPGL might be the limiting factor for positivity on PET or treatment efficacy of somatostatin peptide receptor-based radionuclide therapy (PRRT) [14]. A small number of studies, mainly performed in GEP-NETs, have explored the relationship between SSTR expression of the cell and imaging characteristics in [^68^Ga]Ga-DOTATOC/-DOTATATE PET/CT [15–22]. Overall, a significant correlation of cellular SSTR2 expression and, to a lesser extent, SSTR5 expression, with PET uptake using [^68^Ga]-labeled SSA was found [15–22]. However, whether SSTR expression on the PPGL cell surface correlates with radiolabeled SSA uptake in PET imaging remains unclear. This study examines whether SSTR2 and SSTR5 expression, assessed by both immunohistochemistry and mRNA analysis in patient samples, correlates with radiolabeled SSA uptake in corresponding lesions on [^68^Ga]Ga-DOTATATE PET/CT.

## Methods

### Study population

For this retrospective study, consecutive patients with PPGLs participating in the *ENSAT* registry that were treated at University Hospital Zurich between 2011 and 2024 were included. Inclusion criteria were availability of SSA-coupled radionuclide diagnostic imaging before surgical resection of the PPGL, or systemic treatment in case of metastatic disease. The study was conducted according to regulations of the local Ethics Commission of Zurich under the reference number BASEC 2017–00771 as part of *ENSAT* (*European Network for the Study of Adrenal Tumors*) in accordance with the principles of the Declaration of Helsinki. Written informed consent was obtained from each patient prior to participation.

In patients who agreed to genetic testing, germline and, for some patients, somatic sequencing by next-generation sequencing (NGS) was performed, as previously described [8].

Plasma metanephrines were measured before tumor resection by liquid chromatography–tandem mass spectrometry (LC–MS/MS). Adrenergic PPGLs were defined by plasma metanephrine levels above the reference range and a metanephrine increase exceeding 5% of the total increase of all catecholamines [23]. Noradrenergic PPGLs were defined by a normetanephrine increase with no or small increase of metanephrines. Dopaminergic tumors were defined by isolated increases in 3-methoxytyramine, whereas silent PPGLs showed no elevations of any metanephrines.

### Immunohistochemistry

Immunohistochemistry (IHC) was performed on formalin-fixed, paraffin-embedded (FFPE) 4 μm sections of tumor tissue. Sections were stained at the Department of Pathology, University Hospital Zurich, using the following antibodies: anti-SSTR2A (Cell Marque Lifescreen Ltd.; Cat. No. 486R-16, incubation time 32 min, dilution 1:25), anti-SSTR5 (Zytomed Systems, Cat. No. RBK051-05, incubation time 60 min, dilution 1:5), and anti-carbonic anhydrase IX (anti-CAIX: Abcam Limited Cat. No. 15086, incubation time 30 min, dilution 1:3000). CAIX is a marker of chronic activation of hypoxia-inducible pathways. Staining was carried out using automated platforms (Discovery Ultra, Roche Ventana for SSTR2/5 and BOND Platform for CAIX) and visualized with the OptiView DAB kit for SSTR2/5 and IHC-Refine for CAIX. IHC was manually evaluated by an expert pathologist (UM, hereafter referred to as “manual IHC”) counting the percentage of SSTR2, SSTR5 and CAIX positive cells, where positivity was determined by positive staining (cytoplasmatic or membranous) in at least 1% of tumor cells. For further comparison, SSTR2 positivity was defined as the percentage of SSTR2-positive cells relative to the total number of cells. SSTR5 and CAIX were compared to other variables using a positive (any percentage of positive cells) versus negative (0% positive cells) comparison.

### Digital image analysis (DIA)

Immunohistochemically stained slides for SSTR2 were further analyzed using digital pathology software *QuPath* (version 0.4.3) [24], hereafter referred to as DIA-derived SSTR2 expression. Slides were scanned with a Panoramic 250 scanner (3DHistec) and imported into QuPath. For each tumor, three (in two cases two) representative regions of interest (ROIs) of approximately 1 mm^2^, excluding areas of necrosis, vessels, and stromal artifacts, were selected with the polygon annotation. Automated cell detection was performed within each ROI using QuPath’s built-in algorithm, followed by positive cell detection based on DAB optical density (OD) thresholds to classify each cell into four staining categories: negative, weak (1 +), moderate (2 +), or strong (3 +). Thresholds were manually optimized to match the visual appearance of membranous SSTR2 staining. A histological score (H-score) was calculated per ROI using the formula: (1 × %weak) + (2 × %moderate) + (3 × %strong). The H-score ranges from 0 (all cells negative) to 300 (all strongly positive). To provide an area-normalized measure of SSTR2 expression, we also computed an intensity per area score, defined as: number of cells times mean cell DAB OD divided by ROI area in pixels​. This metric captures the cumulative DAB signal intensity normalized by tissue area and enables quantitative comparison across samples.

### Quantitative reverse transcription PCR (qRT-PCR)

MRNA expression analysis was performed in RNA extracted from two to three cores punched from the FFPE tumor block using the Maxwell RSC RNA FFPE Kit (Promega, Cat. No. AS1440). RNA concentrations were measured with a SpectraMax spectrofluorometer (Molecular Devices), and 1 µg of RNA was reverse transcribed using the iScript™ cDNA Synthesis Kit (Bio-Rad). Quantitative real-time PCR was conducted on a Lightcycler 96 PCR System (Roche) in triplicate. Primer sequences were as follows:

- SSTR2: forward 5′-GGCATGTTTGACTTTGTGGTG-3′, reverse 5′-GTCTCATT CAGCCGGGATTT-3′;
- SSTR5: forward 5′-CTGGTGTTTGCGGGATGTT-3′, reverse 5′-GAAGCTCTGGCGGAAGTTGT-3′;
- 18S rRNA: forward 5′-GGGAGCCTGAGAAACGG-3′, reverse 5′-GGGTCGGGAGTGGGTAATTT-3′.

Gene expression was normalized to 18S rRNA, and relative expression levels were calculated using the ΔCt method.

### Imaging

All patients underwent [^68^Ga]Ga DOTATATE-PET in accordance with the EANM procedure guidelines for PET imaging [25]. The injected tracer activity was 135.5 ± 18.4 MBq of [^68^Ga]Ga DOTATATE (range 101–209 MBq). After an uptake time of 60 min and following CT acquisition for attenuation correction (from the vertex of the skull to the mid-thighs), PET data were acquired in time-of-flight mode, covering the same anatomical region as the CT, with 2–3 min/bed position and 6–8 bed positions per patient (23% overlap). For PET image reconstruction, Bayesian penalized likelihood reconstruction (BSREM—Q.Clear; GE Health Care, Waukesha) was used with a β-value of 1000. Metabolic tumor volume (MTV) was assessed semi manually using an SUV-threshold of 5.0. Organs with physiological uptake were excluded manually from the three-dimensional volume of interest (VOI) covering the entirety of the lesions. SUVmax and SUVmean of tumors was recorded as well as the standard deviation within the VOI of the metabolic tumor volume. Background activity was assessed using the the SUVmean of a 3 cm cubical VOI placed in non-tumorous areas of the liver avoiding central vasculature. To adjust for inter-individual variation, the tumor-to-liver SUV ratios were calculated: SUV_mean_ (tumor SUVmean/liver SUVmean) and SUV_max_ (tumor SUVmax/liver SUVmean). Total lesion uptake (TLU) was defined as rSUV_mean_ multiplied by MTV. SSTR2 positivity in [^68^Ga]Ga DOTATATE-PET was defined as the intensity of uptake in sites of disease that exceeds the normal liver corresponding to a Krenning score equal or greater two [26, 27].

### Statistics

Categorical variables were compared with Pearson's chi-square test or Fisher's exact test, as appropriate. Continuous variables are presented as median (first quartile, third quartile) calculated using Tukey’s hinges method. Variables were assessed for normality by visual inspection and with the Shapiro–Wilk test. For group comparisons, in the case of normally distributed data, one-way ANOVA with Tukey’s post hoc test was used, whereas for non-normally distributed data, the Kruskal–Wallis test with the post hoc Dunn test was applied. Correlation between non-normally distributed IHC, qRT-PCR and [^68^Ga]Ga-DOTATATE PET/CT metrics was assessed using Spearman’s rank correlation. Multivariable linear regression was used to assess associations between PET uptake and SSTR2 IHC expression. Receiver operating characteristic (ROC) analyses were performed to evaluate PET uptake parameters for predicting histological SSTR2 positivity, and optimal cutoffs were derived using the Youden index.

All reported p-values are two-sided hypothesis tests conducted at the p < 0.05 level. Statistical analyses were performed using IBM SPSS Statistics 28.0 (IBM Corporation, Armonk, NY, USA) and the open-source statistics software R (version 4.3.2, R Foundation for Statistical Computing, Vienna, Austria) [28].

## Results

A total of 35 patients, including 19 females (54.3%), were included in this study. Ten patients presented with pheochromocytoma, 20 patients with one or more paragangliomas (16/20 with a head and neck paraganglioma (HNPGL)) and 5 with metastatic disease. Tissue from 38 lesions was available for analysis by IHC and from 22 lesions for qRT-PCR analysis. [^68^Ga]Ga-DOTATATE PET/CT imaging metrics were available for 39 lesions. All patient characteristics are summarized in Table 1, with individual data provided in Supplementary Table 1.

**Table 1:** Patient characteristics

No. of patients	35	
Female (n)/male (n)	19/16	
Age, Median (min, max)	47 (24–84)	
Location		
Pheochromocytoma (n)	10	
Paraganglioma abdominal/thoracic (n)	4	
HNPGL (n)	14	
HNPGL and abdominal paraganglioma (n)	2	
Metastatic disease (n)	5 (PHEO: 2; PGL: 3)	
Metanephrine		
Adrenergic (n)	5	
Noradrenergic (n)	14	
Dopaminergic (n)	3	
Silent (n)	13	
Tissue *(lesion level)*		
Analyzed tumor lesions by IHC (n)	38	
Analyzed tumor lesions by PCR (n)	22	
Analyzed tumor lesions by imaging (n)	39	
Time interval between PET acquisition and tumor resection, months (median, Q1-Q3)	2.1 (0.9, 4.8)	
Pathogenic variant		
Localization	**PHEO/abdominal PGL**	**HNPGL**
Germline		
* SDHB*	3	5
* SDHA*	-	1
* SDHC*	-	2 (1 add. abdominal PGL)
* SDHD*	-	1 (add. abdominal PGL)
* NF1*	2	-
* MAX*	-	1
Negative	9	3
No germline or somatic testing	5	3
Somatic		
* ATRX*	2 (germline negative)	-
* SDHD*	1 (germline negative)	-
* HRAS*	1 (germline negative)	-

*HNPGL* head and neck paraganglioma; *IHC* immunohistochemistry; *PHEO* pheochromocytoma; *PGL* paraganglioma

SSTR2 expression by manual IHC, defined as any tumor cell positivity as determined by the pathologist, was detected in 36 of 38 (95%) tumor samples, whereas only 1/38 (3%) PPGL (a pheochromocytoma) stained positive for SSTR5 by IHC. Any CAIX IHC staining was present in 13/38 (34%) PPGLs (HNPGLs: 47%; pheochromocytomas: 30%; abdominal paragangliomas: 22%).

SSTR2 staining by IHC was further analyzed with DIA software QuPath. DIA H-Score as well as DIA intensity per area showed strong correlation with SSTR2 expression by IHC as scored by the pathologist (r_s_ 0.861 resp. r_s_ 0.695; *p* = 3.38 × 10^–11^ resp. *p* = 3.66 × 10^–6^) (Table 2).

**Table 2:** Correlation between SSTR2 immunohistochemistry (IHC) evaluated manually and with digital image analysis (DIA) and SSTR2 mRNA expression

	SSTR2 expression by IHC (manually evaluated)	SSTR2 mRNA expression
DIA SSTR2 expression: H-Score	rₛ = 0.861, *p* = 3.38 × 10^−11^	rₛ = 0.05, *p* = 0.82
DIA SSTR2 expression: Intensity per area	rₛ = 0.695, *p* = 3.66 × 10^−6^	rₛ = 0.21, *p* = 0.37
SSTR2 expression by IHC (manually evaluated)		rₛ = −0.01, *p* = 0.98

rₛ correlation coefficient

**Fig. 1:**
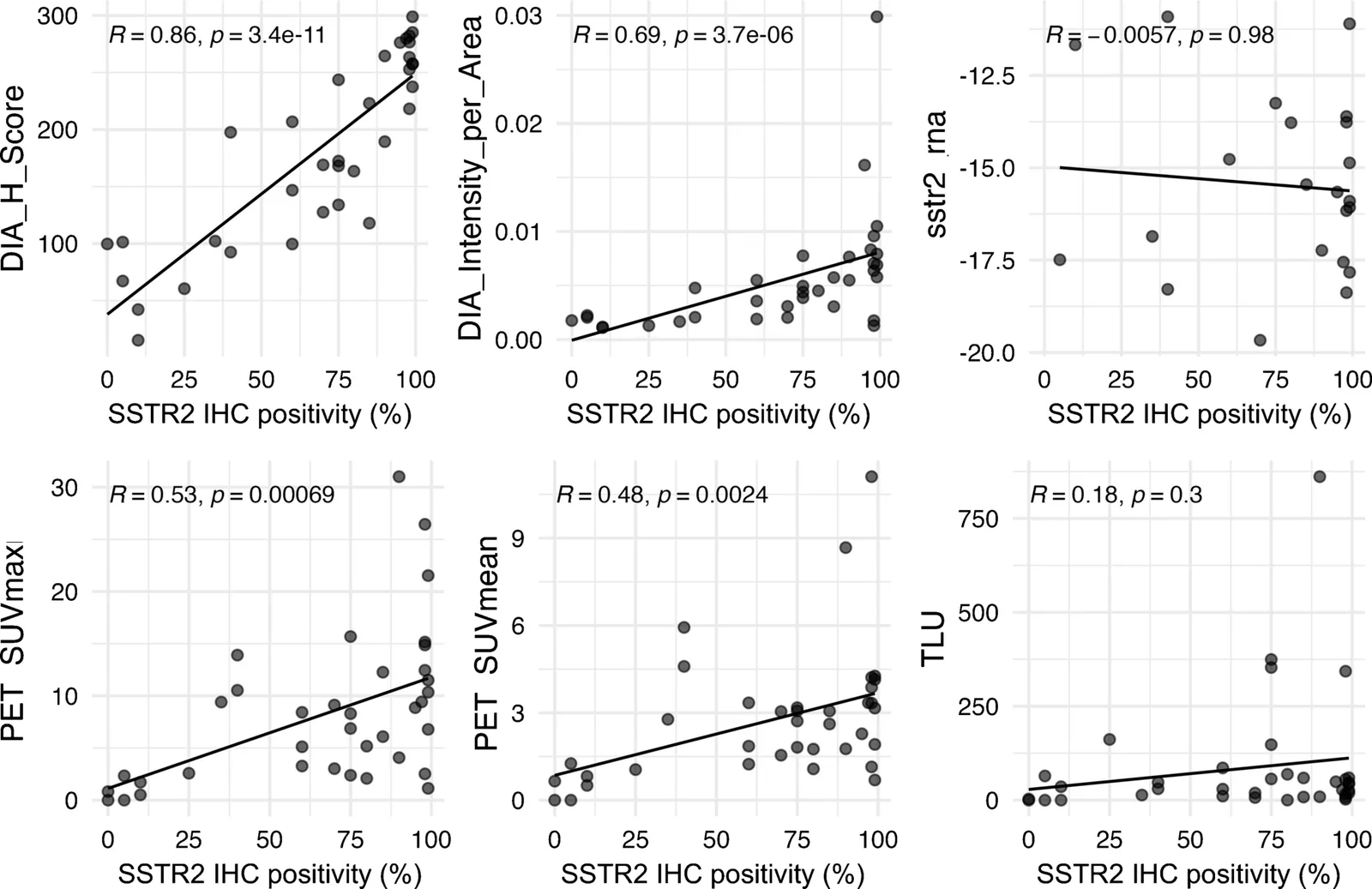
Correlation between SSTR2 expression assessed by immunohistochemistry (IHC), digital image analysis (DIA), SSTR2 mRNA expression, and PET imaging parameters. (A–C) Correlation of manually assessed SSTR2 IHC positivity with DIA-derived SSTR2 H-score, DIA-derived SSTR2 intensity per area, and SSTR2 mRNA expression (− ΔCt). (D–E) Correlation of SSTR2 IHC positivity with liver-corrected [⁶⁸Ga]Ga-DOTATATE PET/CT SUVmax and SUVmean. Correlation coefficients are Spearman’s rₛ

In contrast, the amount of SSTR2 mRNA detected by qRT-PCR did not correlate with IHC SSTR2 expression scored by the pathologist (r_s_ −0.01; p: n.s.), nor with SSTR2 expression determined by DIA (H-Score: r_s_ 0.05; p: n.s.) (Table 2, Fig. 1).

Spearman correlation analysis was then performed to assess the correlation between PET imaging metrics (SUV_max_, SUV_mean_, and TLU) and SSTR2 expression by IHC and SSTR2 mRNA levels. For 37 tumor samples, both IHC and matched PET imaging data from the corresponding lesion prior to resection were available. A statistically significant correlation between SSTR2 expression by manual IHC and both PET lesion SUV_max_ (r_s_ 0.53, *p* = 0.001) and PET lesion SUV_mean_ (r_s_ 0.48, *p* = 0.002) was observed. Similarly, there was a strong correlation between SSTR2 expression as determined by DIA and SUV_max_ (rₛ = 0.54, *p* = 0.001) and SUV_mean_ (rₛ = 0.47, *p* = 0.006) (Table 3, Fig. 1). In contrast, SSTR2 mRNA expression (available from 22 lesions) showed no correlation with any PET metrics. Since most tumors were SSTR5 negative as determined by IHC and qRT-PCR (data not shown), no correlation with PET imaging metrics could be established.

**Table 3:** Correlation SSTR2 expression by immunohistochemistry (IHC) and SSTR2 mRNA expression with PET parameters

	PET: lesion SUV_max_	PET: lesion SUV_mean_	PET: TLU
SSTR2 (% positive cells)	rₛ = 0.532, *p* = 0.001	rₛ = 0.483, *p* = 0.002	rₛ = 0.175, *p* = 0.299
DIA SSTR2 expression: H-Score	rₛ = 0.538, *p* = 0.001	rₛ = 0.466, *p* = 0.006	rₛ = 0.102, *p* = 0.565
DIA SSTR2 expression: Intensity per area	rₛ = 0.466, *p* = 0.005	rₛ = 0.423, *p* = 0.013	rₛ = 0.150, *p* = 0.398
SSTR2 mRNA expression	rₛ = −0.042, *p* = 0.85	rₛ = −0.185, *p* = 0.41	rₛ = 0.086, *p* = 0.702

SUV_mean_ is defined by tumor SUVmean/liver SUVmean and SUV_max_ by tumor SUVmax/liver SUVmean. Total lesion uptake (TLU) was defined as SUV_mean_ * metabolic tumor volume (MTV). DIA: digital imaging analysis; rₛ: correlation coefficient

Expression of SSTR2 and CAIX (indicator of chronic activation of hypoxia-inducible pathways) by IHC, SSTR2 mRNA expression, and PET imaging metrics were compared across different hormonal secretory phenotypes and anatomical location of the PPGLs. The groups of silent and dopaminergic tumors were combined for this analysis due to their shared clinically silent behavior and the low number of dopaminergic cases (*n* = 3).

**Fig. 2:**
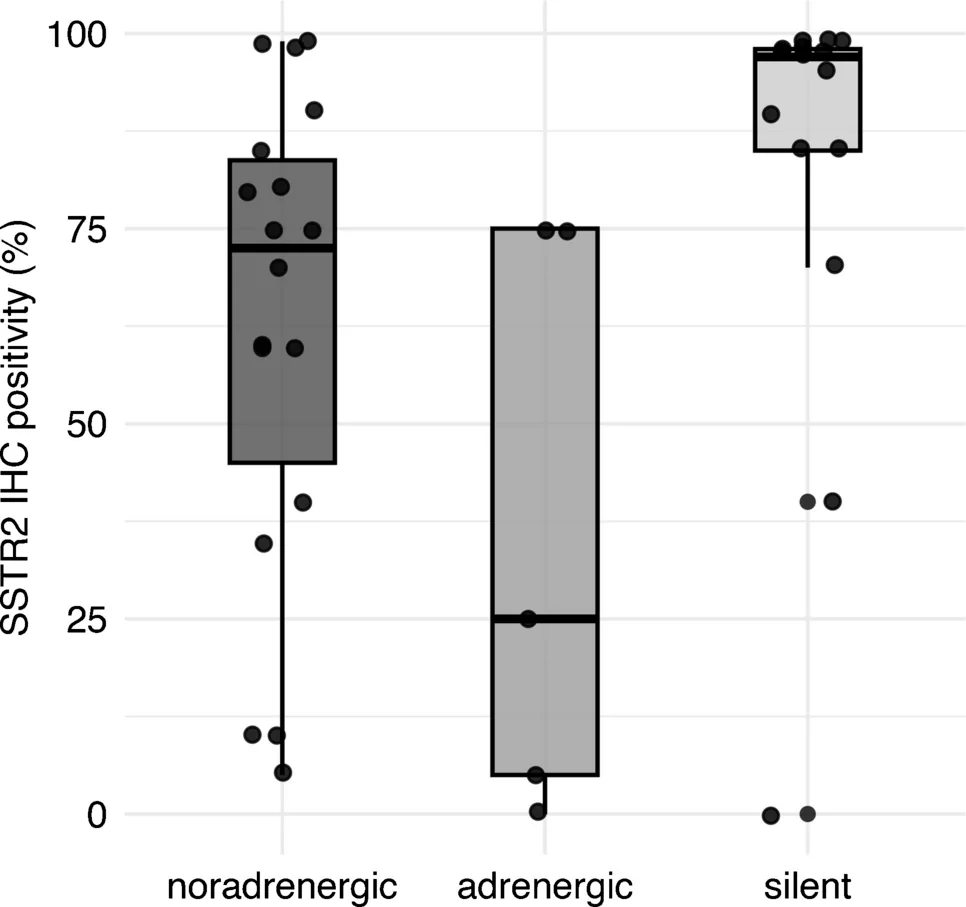
Boxplot showing SSTR2 expression by immunohistochemistry (IHC) assessed manually (% positive tumor cells) across secretory phenotypes. Silent and dopaminergic tumors were combined due to their shared clinically silent behavior and the low number of dopaminergic cases

Silent PPGLs had higher median percentage of SSTR2-positive cells by manual IHC (97%; Q1-Q3: 85–98%) as compared to adrenergic (median 25%; Q1-Q3: 2.5–75%) or noradrenergic PPGLs (median 73%; Q1-Q3: 39–86%) (*p* = 0.009) (Table 4, Fig. 2). Consistently, DIA also revealed a significant difference of SSTR2 expression by IHC between non-secretory and secretory phenotypes, with the highest H-score in silent PPGLs (p = 0.007). There were no significant differences in SUV_mean_, SUV_max_, TLU, mRNA expression or CAIX expression by IHC across PPGLs with different secretory phenotypes (Table 4).

**Table 4:** Comparison of SSTR2 expression by immunohistochemistry (IHC), SSTR2 mRNA expression by qRT-PCR (reported as − ΔCt), and PET-derived metrics across the entire cohort and stratified by hormonal secretory phenotype

	Total	Adrenergic	Noradrenergic	Silent + Dopamineric	*p*
Manual SSTR2 IHC positivity (%)	78 (40–98)	25 (2.5 − 75)	73 (39 − 86)	97 (85 − 98)	0.009
DIA SSTR2 H-Score	189 (102–258)	101 (80 − 189)	163 (96 − 215)	258 (208 − 276)	0.007
DIA SSTR2 expression intensity	0.005 (0.002–0.007)	0.002 (0.002 − 0.006)	0.004 (0.002 − 0.006)	0.006 (0.003 − 0.008)	0.22
SSTR2 mRNA expression (-∆Ct)	−15.8 (−17.5 − −13.7)	−15.4 (−17.5 − −13.3)	−14.8 (−16.6 − −13.8)	−16.1 (−17.8 − −14.1)	0.65
Manual CAIX IHC, positive tumors, n (%)	13 (34%)	2 (40%)	4 (22%)	7 (47%)	0.33
PET lesion SUV_mean_	2.3 (1.1–3.3)	1.3 (0.9 − 3.1)	1.8 (1.0 − 2.8)	3.25 (1.7 − 4.3)	0.058
PET lesion SUV_max_	6.8 (2.5–11.5)	2.6 (1.6 − 7.6)	4.6 (2.0 − 9.2)	10.9 (3.8 − 14.6)	0.036
PET TLU	30 (11–64)	148 (34 − 268)	22 (8 − 60)	45 (11 − 58)	0.18

A total of 38 lesions had IHC data and 39 had imaging data. For imaging analyses by secretory subtype, the distribution was adrenergic (*n* = 5), noradrenergic (*n* = 18), and silent/dopaminergic (*n* = 16). CAIX expression is presented as number (%), whereas all other variables are reported as median (Q1–Q3)

**Fig. 3:**
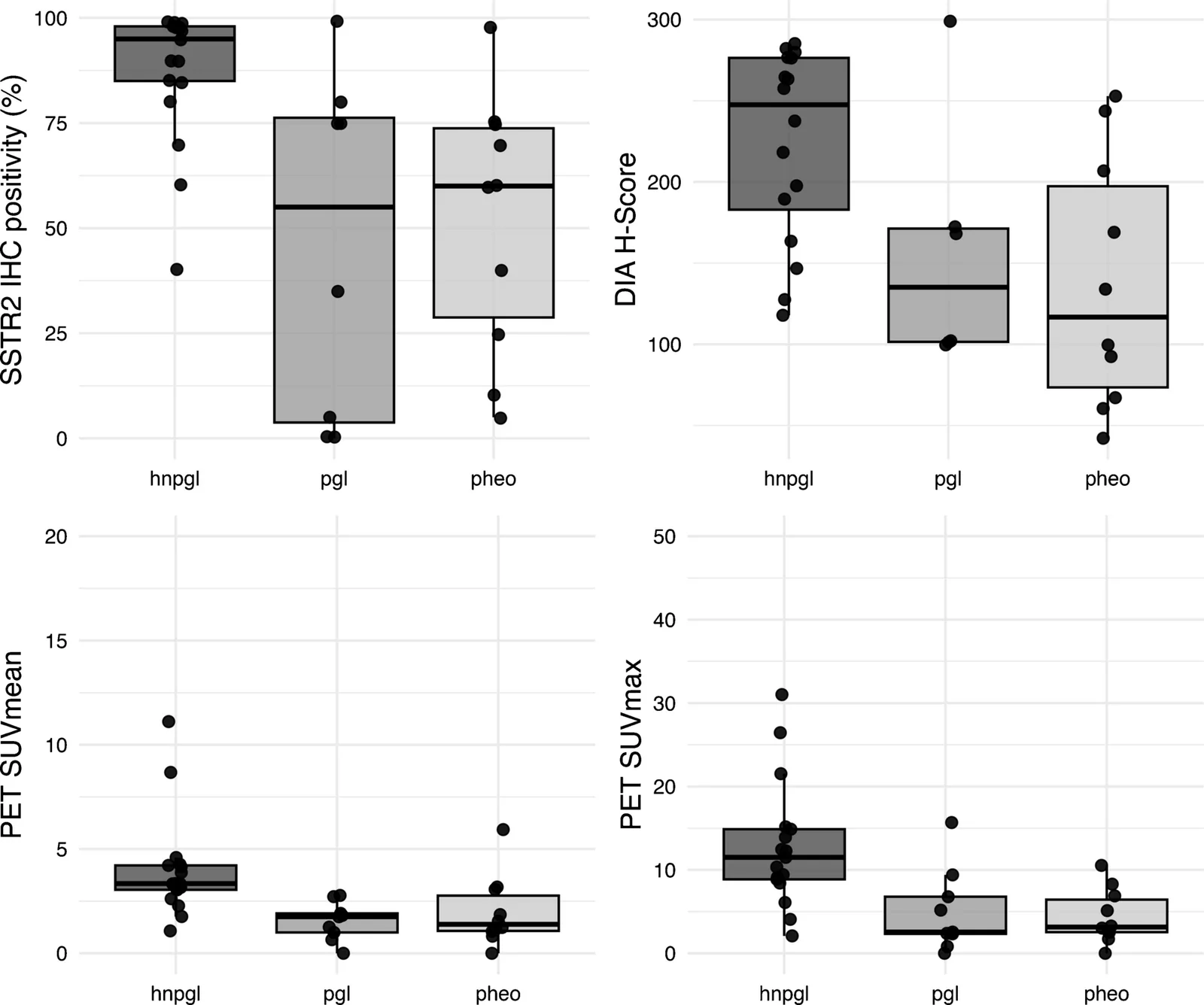
Boxplots showing expression of SSTR2 by immunohistochemistry (IHC) manually assessed and by digital image analysis (DIA) and PET parameters (liver-corrected PET mean SUV and max SUV) across anatomical locations of PPGLs. Abbreviations: paraganglioma (pgl), head and neck paraganglioma (hngpl), pheochromocytoma (pheo)

Significant differences in SSTR2 IHC as well as PET metrics were found across anatomical location of PPGLs. For this analysis, metastatic lesions were excluded due to heterogenicity. SSTR2 expression by IHC (manually and by DIA) was significantly higher in HNPGLs (median 95%; IQR 83–98%) as compared to pheochromocytomas (median SSTR2 expression: 60%; IQR: 21–75%) or abdominal paragangliomas (median SSTR2 expression: 55%; IQR: 1–79%) (*p* = 0.003) (Table 5, Fig. 3). This difference was also reflected by SSA uptake in [^68^Ga]Ga-DOTATATE PET/CT with a median SUV_mean_ of 3.43 (IQR: 2.83–4.24) in HNPGLs as compared to a median SUV_mean_ of 1.75 (IQR: 0.82–2.3) in abdominal paragangliomas and a median SUV_mean_ of 1.39 (IQR: 1–3.1) in pheochromocytomas (*p* = 0.001). Similarly, median SUV_max_ was highest in HNPGLs (median 11.5; IQR: 8.6–15.0) as compared to abdominal paragangliomas (median 2.6; IQR: 1.6–8.1) and pheochromocytomas (median 3.2; IQR: 2.3–7.2)) (*p* = 0.002). Across genotypes, no significant differences in SSTR2 expression or PET metrics were found.

**Table 5:** Comparison of SSTR2 expression by immunohistochemistry (IHC), SSTR2 mRNA expression by qRT-PCR (reported as − ΔCt), and PET-derived metrics across anatomical locations of PPGL

	PHEO	PGL Abdominal	HNPGL	*p*
Manual SSTR2 IHC positivity (%)	60 (21 − 75)	55 (1 − 79)	95 (83 − 98)	0.0026
DIA SSTR2 H-Score	117 (66 − 216)	135 (101 − 204)	248 (170 − 277)	0.013
DIA SSTR2 expression intensity	0.002 (0.001 − 0.004)	0.003 (0.002 − 0.01)	0.006 (0.004 − 0.008)	0.013
SSTR2 mRNA expression (-∆Ct)	−14.77 (−18.98 − −12.46)	−16.38 (−17.33 − −14.31)	−15.86 (−17.5 − −13.65)	0.93
Manual CAIX IHC, positive tumors, n (%)	3 (30%)	2 (22%)	8 (47%)	0.49
PET lesion SUV_mean_	1.39 (1 − 3.1)	1.75 (0.82 − 2.32)	3.34 (2.83 − 4.24)	0.001
PET lesion SUV_max_	3.15 (2.32 − 7.23)	2.55 (1.58 − 8.09)	11.51 (8.64 − 15.01)	0.002
PET TLU	33 (10 − 151)	26 (8 − 67)	43 (9 − 57)	0.92

Available data comprised 10 pheochromocytomas, 9 abdominal paragangliomas, and 17 HNPGLs. CAIX expression is presented as number (%), whereas all other variables are reported as median (Q1–Q3). *HNPGL* head and neck paraganglioma; *PHEO* pheochromocytoma; *PGL* paraganglioma

In multivariable linear regression restricted to primary PPGL lesions and adjusting for tumour site, biochemical phenotype, and the time interval between PET imaging and surgery (log-transformed), SSTR2 positivity by manual IHC showed a positive association with SUV_max_ (β = 0.0132 on the log scale per 1% positivity), corresponding to an estimated 14% higher SUV_max_ per 10 percentage point increase in SSTR2 positivity (*p* = 0.02).

**Fig. 4:**
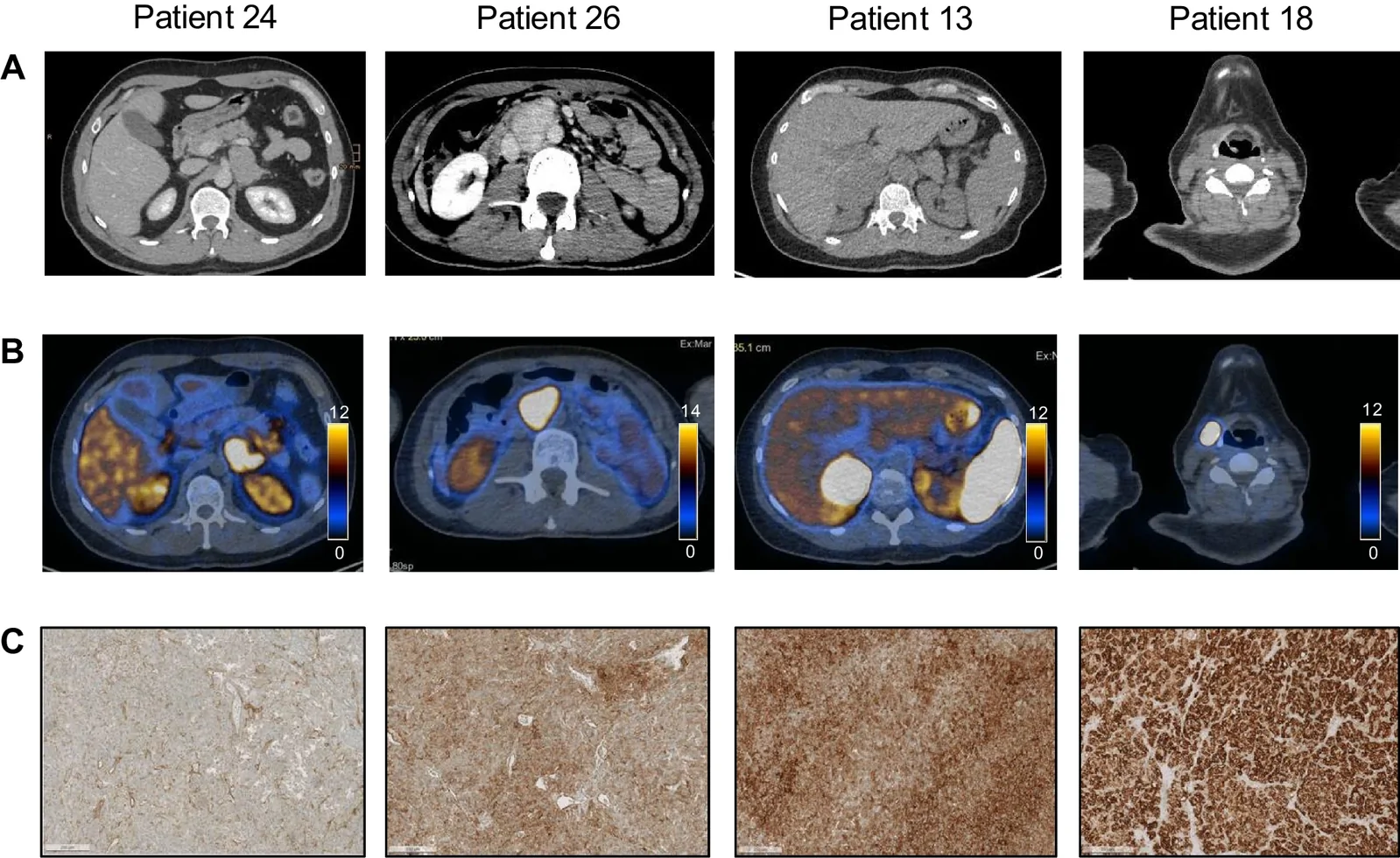
Representative lesion images from four patients illustrating SSTR2 expression by immunohistochemistry (IHC) and corresponding [⁶⁸Ga]Ga-DOTATATE PET/CT findings. Rows depict (A) transverse CT images, (B) transverse PET/CT fusion images demonstrating the PPGL lesions, and (C) corresponding histological sections with SSTR2 IHC staining. Columns represent individual patients (from left to right): Patient 24, periadrenal paraganglioma (Krenning score positive, absolute values: SUVmax 56, SUVmean 22; Liver corrected: SUV_max_ 2.33, SUV_mean_ 1.26; SSTR2 IHC 5%); Patient 26, abdominal paraganglioma (Krenning score positive, absolute: SUVmax: 19, SUVmean 15; Liver corrected: SUV_max_ 2.4, SUV_mean_ 1.8; SSTR2 IHC 75%); Patient 13, pheochromocytoma (Krenning score positive; absolute: SUVmax: 49, SUVmean 22; Liver corrected: SUV_max_ 6.87, SUV_mean_ 3.07; SSTR2 IHC 75%); and Patient 18, head and neck paraganglioma (Krenning score positive; absolute: SUVmax: 79, SUVmean 34; Liver corrected: SUV_max_ 21.53, SUV_mean_ 4.15; SSTR2 IHC 99%)

As PET uptake metrics may be influenced by lesion size, a sensitivity model additionally adjusting for PET-derived lesion volume (log-transformed) was performed. In this model, SSTR2 positivity remained positively associated with SUV_max_ (β = 0.0118), corresponding to an estimated 12.5% higher SUV_max_ per 10 percentage point increase in SSTR2 positivity (*p* = 0.03). Lesion volume showed a borderline association with SUV_max_ (*p* = 0.08), whereas the time interval between PET imaging and surgery was not associated with SUV_max_ (*p* = 0.79). Representative images of SSTR2 expression by IHC and the corresponding lesions on PET/CT are shown in Fig. 4. No significant differences of TLU or SSTR2 mRNA levels were found across different anatomical locations (Table 5). Tumors expressing CAIX by IHC were more frequent in the subgroup of HNPGLs (8/17, 47.1%) compared to pheochromocytomas and abdominal paragangliomas (30% and 22.2% respectively, ns.). CAIX expression did not correlate with SSTR2 expression as determined by IHC, SSTR2 mRNA levels, or SSTR2 PET metrics.

When considering SSTR2 positivity on imaging defined as the intensity of uptake in sites of disease that exceeds the normal liver [26], 4/39 lesions were negative in [^68^Ga]Ga-DOTATATE PET/CT. A positive SSTR lesion in imaging showed perfect sensitivity for SSTR2 expression by IHC using both thresholds (manual IHC ≥ 25% SSTR2 positive cells: 31/31; DIA H-score > 100: 27/27), while specificity was moderate to low (66.7% [4/6] and 42.9% [3/7], respectively).

Next, ROC analyses were performed to derive pragmatic [^68^Ga]Ga-DOTATATE PET/CT thresholds for histological SSTR2 expression. Using DIA H-score > 100 as definition of histological SSTR2 positivity (DIA H-Score SSTR2 negative: 7 samples), SUV_max_ demonstrated good discrimination (AUC 0.83, 95% CI 0.64–1.00), with a Youden-derived optimal SUV_max_ threshold of 5.60 (sensitivity 70%, specificity 86%). Using manual IHC ≥ 25% SSTR2 positive cells as threshold (manual IHC SSTR2 negative: 6 samples), SUV_max_ showed excellent discrimination (AUC 0.98, 95% CI 0.95–1.00), with an optimal threshold of 2.36 (sensitivity 94%, specificity 100%).

## Discussion

In this cohort of PPGL patients, we found a moderate to strong correlation between SSTR2 expression by IHC and [^68^Ga]Ga-DOTATATE PET/CT uptake, a relationship that was previously unexplored in PPGLs. A few studies have investigated the relationship of SSTR expression and imaging characteristics in [^68^Ga]Ga-DOTATOC/-DOTATATE PET/CT in gastro-enteropancreatic NETs [15–19, 21, 22]: Gene expression of SSTR2 as well as IHC scores of SSTR2 and SSTR5 correlated well with SUV_max_ [15–18]. In pancreatic NETs, SSTR2 protein expression by IHC correlated only with SUV_max_, whereas SSTR2 mRNA expression correlated well with SUV_mean_ and SUV_max_ [19].

Conversely, our study in PPGLs demonstrates a significant moderate to strong correlation of SSTR2 expression by IHC with SUV_mean_ and SUV_max_ but no significant correlation with SSTR2 mRNA expression. This supports the assumption that only receptors expressed on the cell surface are relevant for PET radiotracer uptake, while the amount of total SSTR2 mRNA does not seem to predict either protein expression or imaging characteristics in PPGLs.

The lack of correlation between SSTR2 IHC and SSTR2 mRNA expression observed in our cohort warrants further evaluation, particularly given the limited availability of mRNA data and the use of FFPE tissue, which may be affected by RNA degradation. While technical factors may partly account for this finding, post-transcriptional regulation of SSTR2, for example via miRNAs such as miR-375 [29] reported to be overexpressed in PPGLs [30], could represent a potential biological explanation.

In contrast to our results in PPGLs, SSTR2 IHC and SSTR2 mRNA have been reported to correlate in neuroendocrine tumors and in pituitary adenomas [31, 32].

[^68^Ga]Ga-DOTATATE PET/CT has a reported sensitivity of 94% to 100% in the diagnosis and screening of PPGLs in general and has demonstrated superiority for the detection of metastatic paragangliomas and *SDHx*-related PPGLs as compared to [^18^F]F-DOPA-PET, [^18^F]-FDG PET or^123^I-meta-iodobenzylguanidine (^123^I-MIBG) scintigraphy [11–14, 33–35].

Another study using [^18^F]SiTATE-PET/CT for detection of PPGL found higher tracer uptake (SUV_mean_ and SUV_max_ normalized to liver background) in PPGLs with germline *SDHx* and, in particular, *SDHB* pathogenic variants compared to sporadic tumors [36]. In contrast, in our cohort there were no significant differences between SUV_mean_ or SUV_max_ across different genotypes. This lack of significance may be attributable to the limited sample size of our study, since we have previously demonstrated in a larger, multicenter cohort, that at least SSTR2 expression at the cellular level is significantly higher in *SDHB*- and *SDHx*-related PPGLs, compared to other PPGLs [8].

Importantly, in our study the highest expression of SSTR2 by IHC was observed in HNPGLs and in non-secretory PPGL, of which a majority were HNPGLs. In line with this, HNPGLs also showed a significantly higher SUV_mean_ and SUV_max_ compared to pheochromocytomas and abdominal paragangliomas.

Furthermore, our findings are relevant for PRRT, which depends on sufficient radionuclide uptake [14]. In vitro studies have shown that epigenetic drugs can upregulate SSTR2 expression and increase radiotracer uptake in mouse pheochromocytoma cells [37] and human NET cell lines [38–40]. While some preclinical in vivo studies [41] and early clinical trials in epigenetic manipulation in NET patients [42] have been negative, other preclinical in vivo data are promising [43] and several clinical trials are currently recruiting [44, 45].

In addition, CAIX IHC positivity, as an indicator of chronic activation of hypoxia-inducible pathways, was also most frequently found in HNPGLs. As we have demonstrated CAIX expression in HNPGLs (47%), pheochromocytomas (30%) and abdominal paragangliomas (22%), our data seem comparable to the current study from Ohmoto et al., reporting CAIX expression in 50% of PPGLs [46]. CAIX might be a potential target for CAIX-targeted radiopharmaceutical therapy of preselected PPGLs, particularly HNPGLs, in the future, as promising translational/early clinical studies have demonstrated in CAIX-expressing renal cell carcinoma [47, 48].

In our cohort the majority of PPGLs were negative for SSTR5 by IHC, whereas SSTR5 expression correlated with PET metrics in gastrointestinal NETs but not in pancreatic NETs [17, 20].

Our results also demonstrate that for SSTR2 expression at a cellular level, DIA correlates well with analysis by experienced pathologists. Strong correlation between DIA and manual IHC assessment by pathologists for SSTR2 and SSTR5 have been demonstrated for pancreatic NETs and growth hormone-producing pituitary adenomas, respectively [49–51]. DIA may enable high-throughput, automated assessment of tissue samples at lower cost with the possibility for machine-learning, which might be especially relevant in research settings. In addition, DIA allows for a more granular quantification of staining pattern, thus enabling a more precise correlation with continuous PET metrics.

Beyond the assessment of manual and DIA-based IHC, another key strength of our study is the assessment of in vivo radiotracer uptake in corresponding tumor lesions. This enables direct correlation of characteristics of resected PPGL tissue with [^68^Ga]Ga-DOTATATE PET/CT metrics, helping to determine if the respective patient tumor is sensitive to SSA-imaging. In addition, our cohort contains PPGLs from various anatomical locations, including a high percentage of HNPGLs. SSTR2 positivity on imaging was originally defined using ^111^In-pentetreotide scintigraphy as tumor uptake equal to or exceeding normal liver activity, corresponding to Krenning scores 2 to 4 [27]. This threshold has subsequently been adopted for [^68^Ga]Ga-DOTATATE PET/CT [26]. However, [^68^Ga]Ga- DOTATATE PET has been shown to yield higher apparent tracer uptake, and consequently higher Krenning scores, than scintigraphy, particularly in lesions ≤ 2 cm [52]. This is of relevance for HNPGLs, which are frequently small. Consistent with this, our analysis demonstrated a sensitivity of 100% for predicting SSTR2 expression by IHC, whereas specificity was limited.

ROC-derived SUV thresholds may serve as pragmatic imaging correlates of clinically relevant SSTR2 expression and could be particularly useful in non-resected disease (e.g. HNPGLs) to non-invasively identify lesions with high likelihood of substantial SSTR2 expression, thereby supporting consideration of SSTR-targeted therapies such as cold somatostatin analogs or PRRT.

Although the correlations between SSTR2 protein expression and PET-derived SUVs were statistically significant, their magnitude was moderate (rₛ ~ 0.54 to 0.61), indicating that PET uptake is an informative but imperfect surrogate of tissue SSTR2 abundance. This is biologically expected because radiotracer uptake reflects not only receptor density but also lesion perfusion, viable tumor fraction, and receptor accessibility and internalization. Lesion characteristics, particularly tumour volume, may also influence PET-derived uptake metrics. PET metrics summarize tracer uptake over a three-dimensional tumor volume that is frequently heterogeneous, whereas immunohistochemical assessment is based on a limited two-dimensional tissue region. Even when the anatomically corresponding lesion is selected, the resected specimen may not capture the PET-defined subregion with the highest tracer uptake, a spatial sampling mismatch that is likely to attenuate correlation strength while preserving a consistent directional association.

Limitations of this study include, first, the relatively small sample size which restricts the statistical power and limits analysis of subgroups. This limitation is, in part, caused by the fact that a high proportion of patients do not undergo functional imaging before resection of the PPGL. Second, in our dataset, few tumours were SSTR2-negative by either IHC or imaging, resulting in small control groups. Hence, ROC-derived thresholds should also be interpreted cautiously and require external validation. Additionally, our cohort includes only a few patients with metastatic disease, which is important since this is the subgroup most likely to undergo PRRT and TLU did not correlate with SSTR2 expression by IHC.

Third, PET/CT scans were not all acquired on the same scanner platform given the long inclusion period. Although lesion uptake was normalized to liver uptake to mitigate inter-scanner variability, heterogeneity in image acquisition, reconstruction parameters, and quantification cannot be excluded and may have introduced measurement variability. Finally, the retrospective design introduces selection bias, as patients with higher pre-test probability for SSTR2-positive disease (e.g., known *SDHB* variant carriers) may have been preferentially referred for [^68^Ga]Ga-DOTATATE PET/CT. This may have led to an enrichment of SSTR2-positive lesions and also contributed to the low number of SSTR2-negative cases. Furthermore, unmeasured confounders, including tumour heterogeneity, prior treatments, and variability in tissue sampling, may have influenced the observed associations.

In conclusion, this study demonstrates a positive association between SSTR2 expression by IHC and [^68^Ga]Ga-DOTATATE uptake in PPGL. Notably, HNPGLs exhibited high SSTR2 expression and correspondingly high tracer uptake, supporting their biological suitability for SSTR-targeted imaging and therapeutic approaches. While these findings suggest that PET-derived metrics may reflect underlying receptor expression in PPGLs, the clinical applicability of imaging-based thresholds remains exploratory and requires validation in larger, prospective cohorts.

## Supplementary Information

Below is the link to the electronic supplementary material.

Supplementary file1 (DOCX 37 KB)

## Data Availability

The datasets generated during and/or analysed during the current study are available from the corresponding author on reasonable request.
