# Supplementary material for: [68Ga]Ga-DOTATATE uptake in pheochromocytoma/paraganglioma predicts SSTR2 expression by immunohistochemistry: Strongest uptake in head and neck paraganglioma
Source: Eur J Nucl Med Mol Imaging. 2026 Apr 28;53(9):5462–74. doi: 10.1007/s00259-026-07897-0 (PMC13653817; doi:10.1007/s00259-026-07897-0)
Supplement: Supplementary file 1 [file 259_2026_7897_MOESM1_ESM.docx]

| **Patient characteristics** | | | | | | | **Histology** | | | | **[^68^Ga]Ga-DOTATATE PET/CT characteristics** | | | |
| --- | --- | --- | --- | --- | --- | --- | --- | --- | --- | --- | --- | --- | --- | --- |
| **Patient** | **Sex** | **Pathogenic Variant** | **Secretory phenotype** | **Metastatic** | **Lesion localization** | **Time between PET/CT and surgery (in months)** | **IHC SSTR2: manual evaluation of % pos. cells)** | **IHC SSTR2: DIA H-Score** | **IHC SSTR5: manual evaluation of % pos. cells** | **IHC CAIX: manual evaluation of % pos. cells** | **Liver-corrected PET-SUVmax** | **Liver-corrected PET-SUVmean** | **TLU** | **SSTR2 positivity** |
| 1 | m | not tested | noradrenergic | no | Pheo | 0.7 | 10 | 42.13 | 0 | 10 | 1.720 | 0.820 | 35.752 | 1 |
| 2 | m | *ATRX* (somatic) | noradrenergic | yes | Pheo | - | 85 | 222.95 | 0 | 0 | - | - | - |  |
| 2 | m | *ATRX* (somatic) | noradrenergic | yes | Lung metastasis of pheo | 5.7 | 10 | 15.19 | 0 | 0 | 0.506 | 0.506 | 0.046 | 0 |
| 3 | m | *SDHB* | noradrenergic | yes | PGL infrapancreatic | 1.1 | 80 | - | 0 | 0 | 5.176 | 1.753 | 68.891 | 1 |
| 3 | m | *SDHB* | noradrenergic | yes | PGL paraaorta | 1.1 | 75 | 168.12 | 0 | 0 | 15.682 | 2.718 | 353.294 | 1 |
| 4 | m | negative | noradrenergic | no | Pheo | 1.3 | 60 | 206.78 | 0 | 0 | 3.273 | 1.236 | 10.756 | 1 |
| 5 | f | not tested | adrenergic | no | Pheo | 0.3 | 25 | 60.45 | 0 | 20 | 2.580 | 1.050 | 161.700 | 1 |
| 6 | m | *NF1* | adrenergic | no | Pheo | 76.1 | 75 | 133.93 | 0 | 0 | 8.294 | 3.176 | 147.706 | 1 |
| 7 | f | negative | dopaminergic | yes | PGL paravertebral | - | - | - | - | - | 2.545 | 1.000 | 22.800 | 1 |
| 8 | f | not tested | noradrenergic | no | Pheo | 0.2 | 60 | 99.57 | 0 | 0 | 5.118 | 1.855 | 85.899 | 1 |
| 9 | m | *SDHB* | noradrenergic | no | HNPGL (Glomus caroticum) | 9.6 | 90 | 189.38 | 0 | 0 | 4.075 | 1.763 | 8.989 | 1 |
| 10 | f | *SDHB* | silent | no | HNPGL (dorsal thyroid) | 2.2 | 98 | 218.20 | 0 | 5 | 26.444 | 11.111 | 343.333 | 1 |
| 11 | m | not tested | noradrenergic | no | Pheo | 0.5 | 40 | 92.46 | 40 | 0 | 10.533 | 5.933 | 30.260 | 1 |
| 12 | f | negative | dopaminergic | no | Pheo | 3.8 | 70 | 168.99 | 0 | 0 | 3.022 | 1.543 | 18.985 | 1 |
| 13 | f | negative | adrenergic | no | Pheo | 0.1 | 75 | 243.63 | 0 | 1 | 6.873 | 3.070 | 374.592 | 1 |
| 14 | f | negative | adrenergic | no | PGL periadrenal | 2.2 | 0 | 99.64 | 0 | 0 | 0.824 | 0.653 | 2.938 | 0 |
| 15 | m | *SDHD* | silent | no | HNPGL (Glomus caroticum) | 4.0 | 40 | 197.57 | 0 | 5 | 13.906 | 4.594 | 47.316 | 1 |
| 16 | f | *ATRX* (somatic) | noradrenergic | yes | PGL retroperitoneal | 3 | 99 | 257.67 | 0 | 0 | 1.133 | 0.692 | 18.883 | 1 |
| 17 | f | *SDHB* | silent | no | HNPGL (Carotid bifurcation) | 2.8 | 97 | 279.87 | 0 | 5 | 9.420 | 3.346 | 27.435 | 1 |
| 18 | f | *SDHB* | silent | yes | HNPGL (submandibular) | 0.5 | 99 | 257.53 | 0 | 80 | 21.530 | 4.145 | 59.682 | 1 |
| 19 | m | *NF1* | noradrenergic | no | PGL periadrenal | 0.9 | 99 | 298.91 | 0 | 0 | 6.786 | 1.914 | 25.843 | 1 |
| 20 | m | *SDHD* | noradrenergic | no | HNPGL (Glomus caroticum right) | 23.9 | 70 | 127.51 | 0 | 0 | 9.119 | 3.045 | 7.003 | 1 |
| 20 | m | *SDHD* | noradrenergic | no | HNPGL (Glomus caroticum left) | 8.7 | 60 | 146.74 | 0 | 0 | 8.418 | 3.343 | 29.421 | 1 |
| 20 | m | *SDHD* | noradrenergic | no | PGL abdominal | 4.6 | 35 | 102.13 | 0 | 0 | 9.403 | 2.776 | 13.603 | 1 |
| 21 | m | *SDHB* | noradrenergic | yes | PGL retroperitoneal | 4.2 | - | - | - | - | 3.571 | 1.264 | 278.022 | 1 |
| 22 | m | *SDHD* (somatic) | noradrenergic | no | HNPGL (Glomus caroticum) | 1.9 | 98 | 282.13 | 0 | 10 | 15.155 | 3.333 | 15.667 | 1 |
| 23 | f | negative | dopaminergic | no | HNPGL (Glomus jugulare) | 1.4 | 90 | 264.54 | 0 | 0 | 31.013 | 8.675 | 861.428 | 1 |
| 24 | m | *HRAS* (somatic) | adrenergic | no | PGL periadrenal | 0.4 | 5 | 101.29 | 0 | 0 | 2.333 | 1.258 | 64.413 | 1 |
| 25 | m | *SDHB* | silent | no | HNPGL (Carotid bifurcation) | 6.0 | 98 | 276.66 | 0 | 0 | 12.452 | 4.215 | 55.639 | 1 |
| 26 | f | *SDHC* | noradrenergic | no | HNPGL (Carotid bifurcation) | 9.2 | 80 | 163.46 | 0 | 10 | 2.086 | 1.074 | 0.215 | 1 |
| 26 | f | *SDHC* | noradrenergic | no | PGL paraaortal | 1.7 | 75 | 172.31 | 0 | 3 | 2.383 | 1.815 | 56.441 | 1 |
| 27 | m | negative | silent | no | Pheo | 0.8 | 98 | 252.83 | 0 | 0 | 2.520 | 1.143 | 7.086 | 1 |
| 28 | f | *SDHB* | silent | no | HNPGL (Glomus caroticum) | 2.9 | 85 | 117.86 | 0 | 0 | 6.089 | 3.063 | 59.122 | 1 |
| 29 | m | not tested | noradrenergic | no | Pheo | 0.9 | 5 | 67.13 | 0 | 0 | 0.697 | 0.385 | 11.154 | 0 |
| 30 | f | *SDHA* | silent | no | HNPGL (Glomus caroticum) | 1.6 | 99 | 285.15 | 0 | 1 | 11.505 | 3.162 | 43.314 | 1 |
| 31 | f | not tested | silent | no | HNPGL (Glomus caroticum) | 1.7 | 99 | 237.48 | 0 | 1 | 10.342 | 4.263 | 46.468 | 1 |
| 32 | f | negative | silent | no | HNPGL (temporal) | 11.0 | 85 | - | 0 | 0 | 12.267 | 2.616 | 8.372 | 1 |
| 33 | f | *MAX* (VUS) | silent | no | HNPGL (Glomus tympanicum) | 2.4 | 98 | 263.35 | 0 | 0 | 14.875 | 3.875 | 2.325 | 1 |
| 34 | f | negative | silent | no | HNPGL (Glomus caroticum) | 5.5 | 95 | 276.27 | 0 | 0 | 8.865 | 2.279 | 48.995 | 1 |
| 35 | f | negative | silent | no | PGL periadrenal | 1.3 | 0 | - | 0 | 60 | 0 | 0 | 0 | 0 |

**Supplementary Table 1.** Individual patient characteristics, including histology and PET/CT parameters. *Abbreviations: head and neck paraganglioma (HNPGL); immunohistochemistry (IHC), pheochromocytoma (Pheo), paraganglioma (PGL).*
